# Physiological and Molecular Responses of ‘Dusa’ Avocado Rootstock to Water Stress: Insights for Drought Adaptation

**DOI:** 10.3390/plants10102077

**Published:** 2021-09-30

**Authors:** Moreno-Ortega Guillermo, Zumaquero Adela, Matas Antonio, Nicholas A. Olivier, van den Berg Noëlani, Elena Palomo-Ríos, Martínez-Ferri Elsa, Pliego Clara

**Affiliations:** 1 Departamento de Genómica y Biotecnología, Fruticultura Subtropical y Mediterránea (IFAPA) Unidad Asociada de I+D+i al CSIC, Cortijo de la Cruz s/n, 21940 Churriana, Spain; guillermo.moreno.o@juntadeandalucia.es (M.-O.G.); zumaquero@uma.es (Z.A.); mclara.pliego@juntadeandalucia.es (P.C.); 2 Departamento de Ecofisiología de Cultivos, Fruticultura Subtropical y Mediterránea (IFAPA) Unidad Asociada de I+D+i al CSIC, Cortijo de la Cruz s/n, 21940 Churriana, Spain; 3 Departamento de Botánica y Fisiología Vegetal, Instituto de Hortofruticultura Subtropical y Mediterránea “La Mayora” (IHSM-UMA-CSIC), Unidad Asociada IHSM-IFAPA, Universidad de Málaga, 29071 Málaga, Spain; antoniojmatas@uma.es (M.A.); epalomorios@uma.es (E.P.-R.); 4 Department of Plant and Soil Science, University of Pretoria, Pretoria 0002, South Africa; nicky.olivier@fabi.up.ac.za; 5 Department of Biochemistry, Genetics and Microbiology, University of Pretoria, Pretoria 0002, South Africa; noelani.vandenberg@up.ac.za; 6 Forestry and Agricultural Biotechnology Institute (FABI), University of Pretoria, Pretoria 0002, South Africa

**Keywords:** *Persea americana*, abiotic stress, water deprivation, microarray

## Abstract

Avocado consumption is increasing year by year, and its cultivation has spread to many countries with low water availability, which threatens the sustainability and profitability of avocado orchards. However, to date, there is not much information on the behavior of commercial avocado rootstocks against drought. The aim of this research was to evaluate the physiological and molecular responses of ‘Dusa’ avocado rootstock to different levels of water stress. Plants were deficit irrigated until soil water content reached 50% (mild-WS) and 25% (severe-WS) of field capacity. Leaf water potential (Ψ_w_), net CO_2_ assimilation rates (*A*_N_), transpiration rate (*E*), stomatal conductance (*g*_s_), and plant transpiration rates significantly decreased under both WS treatments, reaching significantly lower values in severe-WS plants. After rewatering, mild- and severe-WS plants showed a fast recovery in most physiological parameters measured. To analyze root response to different levels of drought stress, a cDNA avocado stress microarray was carried out. Plants showed a wide transcriptome response linked to the higher degree of water stress, and functional enrichment of differentially expressed genes (DEGs) revealed abundance of common sequences associated with water stress, as well as specific categories for mild-WS and severe-WS. DEGs previously linked to drought tolerance showed overexpression under both water stress levels, i.e., several transcription factors, genes related to abscisic acid (ABA) response, redox homeostasis, osmoprotection, and cell-wall organization. Taken altogether, physiological and molecular data highlight the good performance of ‘Dusa’ rootstock under low-water-availability conditions, although further water stress experiments must be carried out under field conditions.

## 1. Introduction

Among all environmental factors representing a threat to agricultural production, drought has the largest impact on crop productivity [1]. Drought occurs in almost all climatic regions, and it induces crop yield loss in a wide range of plants, while also increasing global tree mortality [2]. Predicted scenarios of climate change suggest that regions, such as the Mediterranean basin, which includes the subtropical Andalusian coast, might be especially vulnerable to global warming and drought [3], calling into question the long-term sustainability of certain crops. Therefore, characterizing plants’ responses to water stress is needed to provide insight into drought effects on plants and elucidate the mechanisms to recognize external stress signals that trigger changes from physiological to molecular levels, which finally lead to a decrease in crop yield.

Avocado (*Persea americana* Mill.) is considered one of the most nutritional fruits [4], whose consumption is increasing year by year. Its cultivation has spread to many countries from the tropics to the Mediterranean region, facing low-water-availability environments. In this scenario, the sustainability and profitability of the avocado crop should rely on a better use of water [5] and the selection of drought-tolerant avocado rootstocks. However, to date most of the rootstock selections have focused on root rot and salinity tolerance, and there is not much information on the behavior of commercial avocado rootstocks under drought stress situations. The avocado rootstock ‘Dusa’ was commercially released in 2004 and it has replaced previous selections such as ‘Duke 7’, becoming the most extensively used clonal rootstock worldwide [6]. Well adapted to temperate and subtropical climates, ‘Dusa’ has shown excellent behavior against *Phytophthora cinnamomi* [7], resistance to *Verticillium dahliae* [8], an acceptable tolerance to salinity [9], and graft compatibility with many scions, giving rise to high-yielding combinations [10]. However, to date, there is no report dealing with the physiological and molecular response of ‘Dusa’ to water stress.

Understanding the molecular mechanisms underlying drought stress tolerance has been an active area of research, and recent transcriptome studies have identified many genes related to drought stress responses in plants [11,12,13,14,15,16], including those encoding detoxification enzymes, osmoprotectants, heat-shock proteins (HSPs), and phytohormones. In addition, several genes encoding transcription factors (TFs) that regulate and provide adaptive responses to water stress have been identified such as NAC, WRKY, MYB, and bZIP, and some of them have been engineered to improve stress tolerance in model and crop plants [17,18]. Despite the development of next-generation sequencing leading to considerable progress, the molecular mechanisms underpinning drought tolerance are not yet fully elucidated. In addition, reports in different species point to the existence of both conserved and species-specific drought-inducible genes, suggesting the complex nature of the drought stress response [19].

The aim of this study was to shed light on the mechanisms underlying the response of ‘Dusa’ avocado rootstock to different levels of water stress via a combination of physiological measurements and gene expression analysis. Molecular studies were carried out using an Agilent array specifically designed to study the gene expression profiles of ‘Dusa’ rootstocks subjected to biotic [20,21] and abiotic stresses [20]. Evaluating the response of ‘Dusa’ to different levels of water stress will provide key information for subsequent investigations related to the improvement of water use efficiency and drought tolerance in avocado.

## 2. Results and Discussion

### 2.1. Physiological Response to Drought Stress and Rewatering

**Figure 1:**
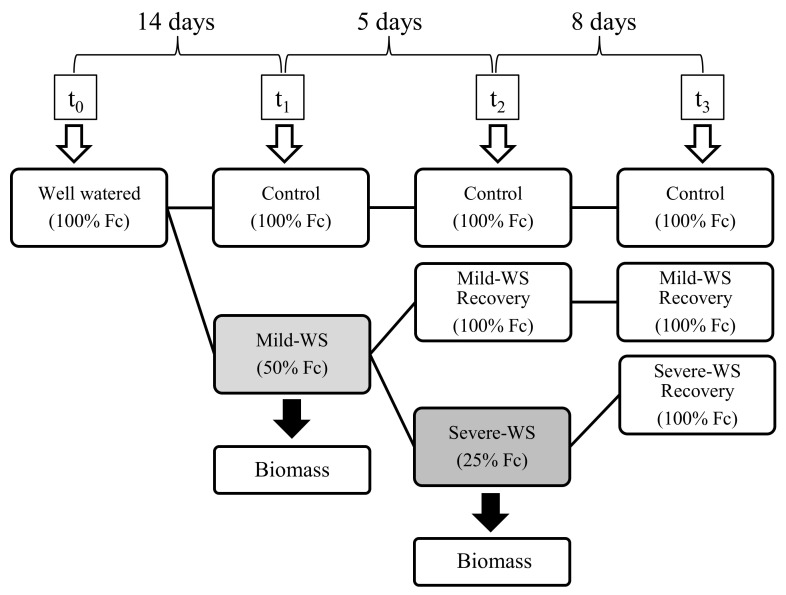
Schematic illustration of the experimental design. Control plants were maintained at field capacity (Fc) throughout the experiment, and water-stressed plants were subjected to substrate drying-up until they reached 50% of Fc (mild-WS; t_1_) and 25% of Fc (severe-WS; t_2_), respectively. Subsequently, plants were fully irrigated to assess the drought recovery response (t_2_ and t_3_).

To investigate the physiological response of ‘Dusa’ rootstock to water stress, plants were deficit irrigated until soil water content (SWC) reached 50% (i.e., mild-WS) and 25% (i.e., severe-WS) of field capacity (Fc). Once these levels were reached, rewatering was carried out, and Fc values were attained immediately (Figure 1). A set of physiological measurements were taken at the leaf and whole-plant levels to assess the response of ‘Dusa’ to each level of water stress and rewatering.

**Figure 2:**
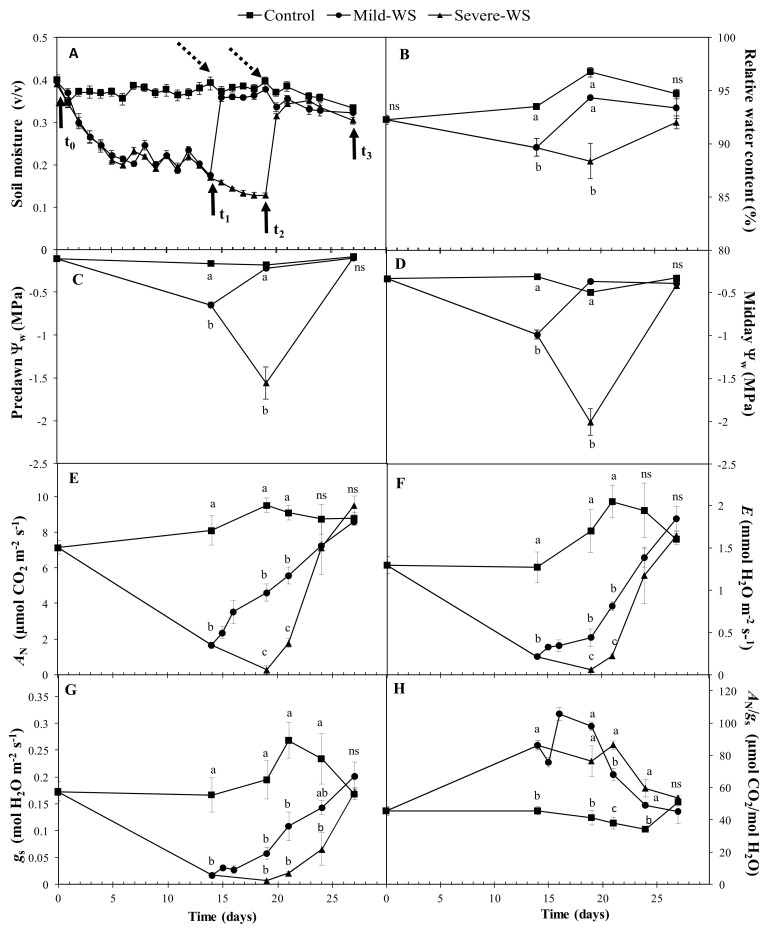
Time-course of mean values (± SE; *n* = 4 to 16) of volumetric soil moisture (**A**), relative water content (**B**), predawn (**C**) and midday leaf water potential (Ψ_w_; **D**), net CO_2_ assimilation rate (*A*_N_; **E**), transpiration rate (*E*; **F**), stomatal conductance (*g*s; **G**), and intrinsic water use efficiency (*A*_N_/*g*_s_; **H**) of ‘Dusa’ plants subjected to three water treatments: control, mild-WS (50% Fc), and severe-WS (25% Fc). Solid arrows indicate the timepoints where measurements and samplings were done, while dotted arrows show rewatering days. Different letters indicate significant differences among treatments for the specific timepoints (days) (*p* < 0.05; ns: no statistical differences).

The soil moisture of water-stressed plants decreased from field capacity (~0.4 *v*/*v*) to ~0.2 *v*/*v* in mild-WS in 14 days (t_1_) and, 5 days later (t_2_), to ~0.1 *v*/*v* in severe-WS (Figure 2A). These changes in SWC were translated into significantly lower values of leaf relative water content (RWC) in mild-WS and severe-WS (89.65% ± 0.81% and 88.37% ± 1.54%, respectively) compared to those of control plants (between 92.25% ± 0.44% and 96.72% ± 0.44%; *p* < 0.05; Figure 2B), but RWC values did not match stress severity. In contrast to RWC, predawn and midday leaf water potential (Ψ_w_) values of water-stressed plants decreased significantly in comparison with control plants (*p* < 0.05) and accordingly with the soil water depletion (Figure 2C,D), supporting the use of this parameter as an appropriate plant-based water stress indicator in avocado [5,22]. Despite both treatments reaching values of predawn and midday leaf water potential indicative of drought stress [23,24,25], water-stressed plants recovered control values within 5–8 days of rewatering, which possibly indicates the robustness of the water transport system in ‘Dusa’ plants. This fast recovery could be related to specific anatomical vessel features associated with the low vulnerability to cavitation reported in Guatemalan and Mexican avocado races [26], from which ‘Dusa’ is a hybrid. This hypothesis is consistent with the relationship between a fast recovery of water potential values with water transport via remaining intact xylem conduits in another woody species [27].

Net CO_2_ assimilation rate (*A*_N_), transpiration rate (E), and stomatal conductance (*g*_s_) showed a marked and significant decrease in both water stress levels (*p* < 0.05; Figure 2E–G). Mild-WS involved a decrease in gas exchange parameters (*A*_N_, *E*, and *g*_s_) of around 80–88% compared to control values, while severe-WS caused a reduction of 95–97%. Stomatal closure to avoid water losses through transpiration and the resulting decrease in assimilation rates are common plant responses to water stress [28,29]. Water-stressed avocado plants were able to maintain certain CO_2_ assimilation rates despite the low stomatal conductance values. This led to higher intrinsic water use efficiency (*A*_N_/*g*_s_) in both levels of water stress compared to control plants, with mild-WS plants displaying higher values (Figure 2H). All gas exchange parameters were recovered up to control values after rewatering regardless of water stress severity, but it is remarkable that severely water-stressed avocado plants recovered even faster than mild-WS ones (5 and 10 days, respectively), which is in contrast with the long-lasting recovery commonly associated with increased levels of water stress [27]. In this sense, it should be noted that, although soil-water depletion in plants with mild-WS was lower than in those with severe-WS, in the former, the level of stress was maintained for 9 days before measurements (Figure 2), which can account for the small differences between the water treatments observed in the response to water stress.

Nevertheless, the faster recovery of *A*_N_ in comparison to *g*_s_, resulting in higher values of *A*_N_/*g*_s_ after rewatering, in both levels of water stress, suggests that the drought-induced decrease in photosynthesis was mainly mediated by stomatal limitations [30,31]. This mismatching response has already been described in other woody species [32,33] and is the basis for suggesting the use of deficit irrigation strategies for increasing crop water use efficiency [34]. It is feasible to conclude that the fast and complete recovery of all gas exchange parameters at both levels of water stress indicate a lack of persistent damage in the photosynthetic apparatus. This is supported by the absence of significant differences in the relative chlorophyll content (SPAD index) indicating that no chlorophyll degradation was associated with any of the water stress treatments (average value of SPAD index was 65.9 ± 0.9 in all treatments) and by the high predawn photochemical efficiency of PSII (*F*_v_/*F*_m_) of all plants regardless of the water treatment (average *F*_v_/*F*_m_ was 0.8 ± 0.04 in all treatments).

**Figure 3:**
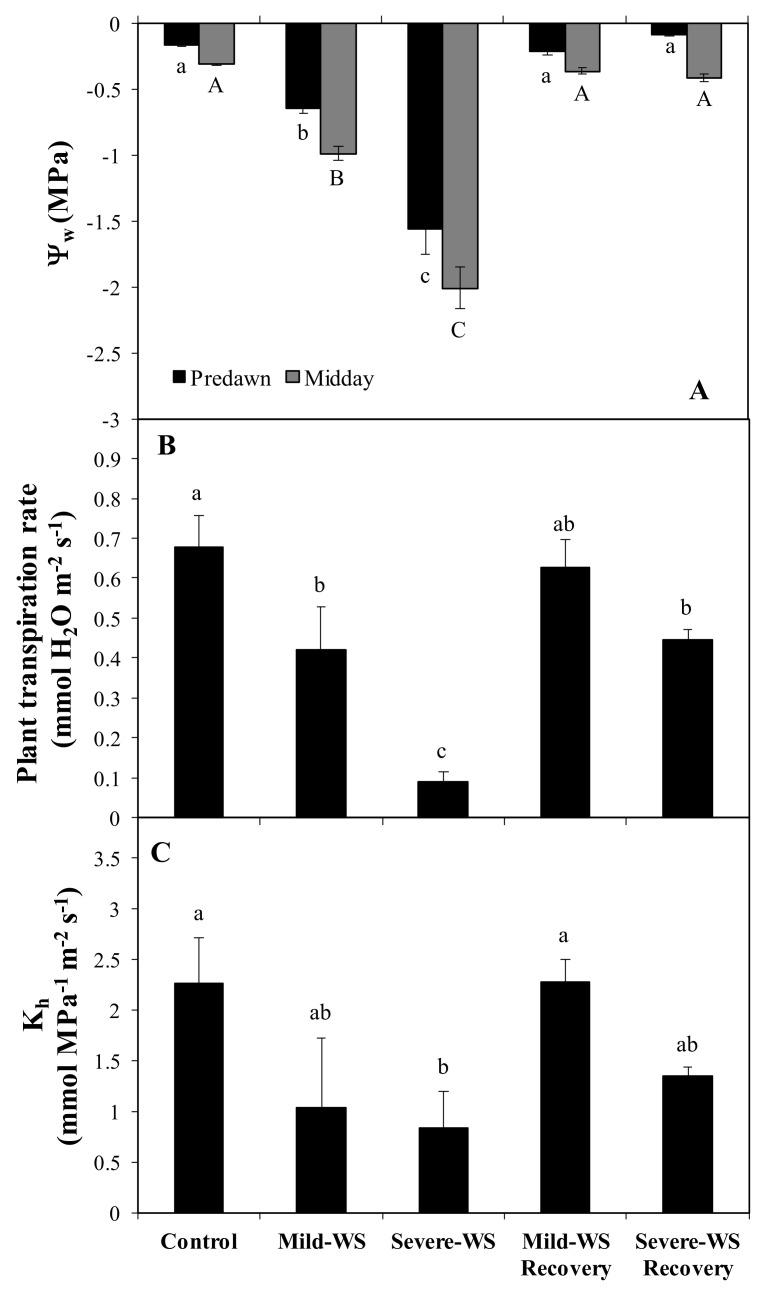
Predawn and midday water potential (Ψw; **A**), plant transpiration rate (**B**), and plant hydraulic conductance (*K*_h_; **C**) from ‘Dusa’ avocado rootstock subjected to two different levels of water stress: mild-WS (50% of Fc) and severe-WS (25% of Fc (mean ± SE; *n* = 4) and their corresponding recovery. Within each series, different capital or lowercase letters indicate significant differences among treatments (*p* < 0.05).

The set of changes at the leaf level in response to water stress (Figure 2, Figure 3A) were accompanied by adjustments at the whole-plant level. Plant transpiration rate was significantly lower (*p* < 0.05; Figure 3B) in both levels of water stress, showing 42% and 86% of control values in mild-WS and severe-WS, respectively. Consistently, plant hydraulic conductance (K_h_) was also affected in both water stress treatments, being significantly lower in severe-WS (*p* < 0.05; Figure 3C). After rewatering, K_h_ recovered near-control values but plant transpiration rate in severe-WS was still significantly lower than that in control plants. Since SWC and leaf water potential were fully restored after rewatering, the persistence of the lower plant transpiration rates could indicate some degree of embolized conduits associated with severe drought stress [35]. In this sense, the significantly higher root amount in water-stressed plants (*p* < 0.05, Table 1) was not enough to counteract the negative effects of severe-WS on K_h_ after rewatering. This suggests that this plant hydraulic impairment might result from disorders either at the trunk or at the root level, since roots are typically more vulnerable than shoots to cavitation, being the weakest link along the hydraulic flow path from soil to atmosphere under drought stress [36,37].

The significantly higher root/shoot ratio (*p* < 0.05, Table 1) observed in mild-WS and severe-WS plants is consistent with previous findings in drought-tolerant genotypes, where an increase in the ratio of root biomass to aerial parts was observed in response to water stress [38]. It is interesting to note that root growth induced by water deprivation occurred in a short period (14 and 19 days in mild-WS and severe-WS, respectively), suggesting a fast adaptative strategy of ‘Dusa’ to cope with drought stress.

### 2.2. Transcriptional Responses of ‘Dusa’ Avocado Rootstock Subjected to Mild and Severe Water Stress

**Figure 4:**
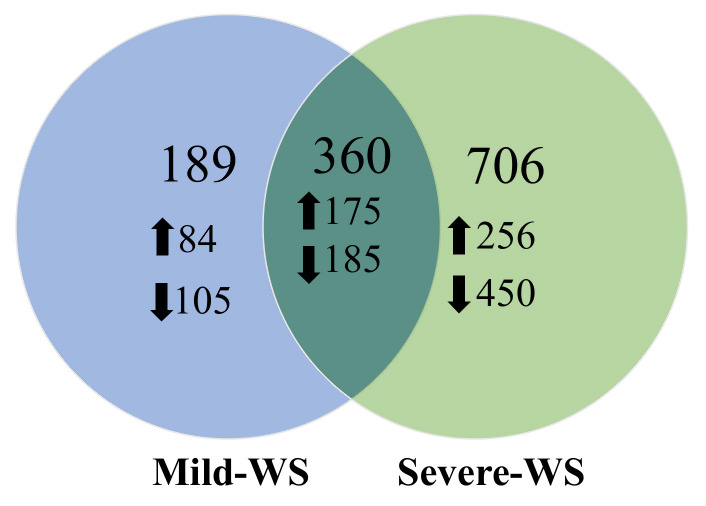
Venn diagram of differentially expressed genes. Numbers of common and specific differentially expressed genes (DEGs) obtained in the microarray analysis of ‘Dusa’ avocado rootstocks subjected to two different levels of water stress (50% of Fc, mild-WS and 25% of Fc, severe-WS). Statistically significant DEGs (*p* < 0.05) were filtered above and below fold change values of 2 and −2, respectively. Unique DEGs are shown only in one of the two circles, while shared transcripts are shown in their intersection. Arrows indicate the number of genes up- and downregulated.

Water stress affects several biological processes, and plants must change their global gene expression patterns to survive water shortage. Since soil water limitation is initially detected by the roots, characterizing the differentially expressed genes (DEGs) in response to different levels of water stress is critical to understand the molecular basis of drought tolerance. To analyze the avocado response to water shortage, a targeted cDNA avocado stress microarray [10] containing transcripts from de novo sequencing of ‘Dusa’ in response to biotic and abiotic stress [20] was used. Root samples were collected at t_1_ and t_2_ corresponding to mild-WS and severe-WS, respectively (Figure 1). The hybridization percentages of the microarrays were similar for the two timepoints, being 78.32% for mild-WS and 76.59% for severe-WS. The total number of DEGs on the array was 549 (47.2% induced and 52.8% repressed) and 1066 (40.4% induced and 59.6% repressed) in mild-WS and severe-WS, respectively (−2 > fold change (FC) > 2; *p* < 0.05). As shown in Figure 4, 189 genes were specific to mild-WS and 706 were specific to severe-WS. The increased number of DEGs observed in severe-WS is in agreement with other transcriptome studies in which a higher degree of water stress involves a wider transcriptome response [12,16]. Regarding the overall response to water deprivation in avocado, there were more downregulated than upregulated genes. Similar results have previously been observed in other crops subjected to drought stress [14,16,39,40,41,42,43,44].

### 2.3. Validation of the Microarray

Differences found in gene expression profiles between mild-WS and severe-WS were further verified by performing a real-time quantitative qPCR (qRT-PCR) assay on total cDNA samples from roots of three biological replicates. Thirteen unigenes showing contrasting expression patterns among mild-WS and severe-WS were analyzed. Negative controls were used to confirm the absence of contamination and actin was used as a reference gene for data normalization. The expression levels of these genes amplified by qRT-PCR are shown in Table 2. The results corroborated the overall differences found among mild-WS and severe-WS in the microarray analysis.

### 2.4. Functional Annotation and GO Term Enrichment Analysis of the Differentially Expressed Genes (DEGs) of ‘Dusa’ Avocado Roots Subjected to Mild and Severe Water Stress

**Figure 5:**
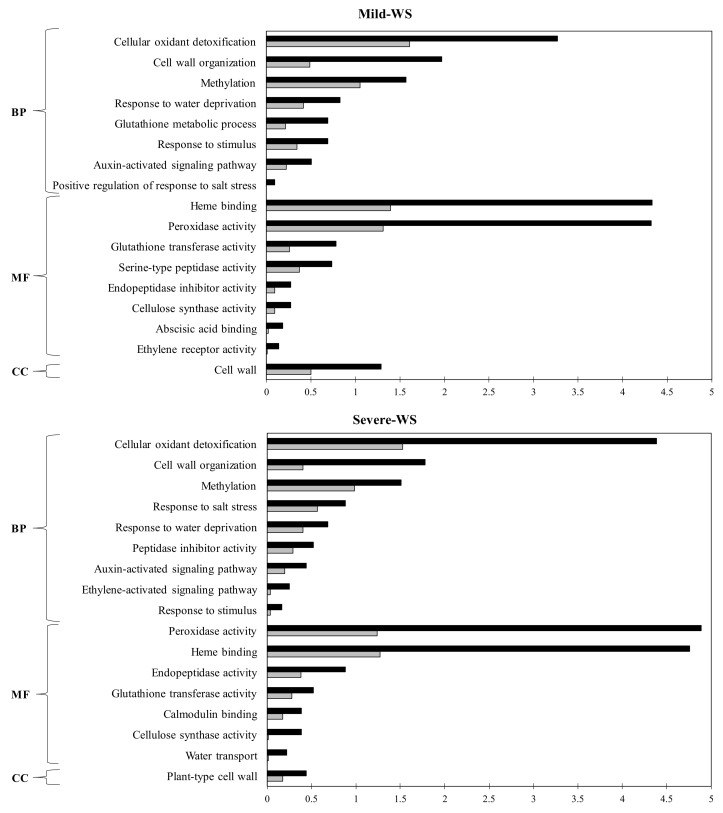
Gene Ontology (GO) enrichment analysis of differentially expressed genes (DEGs) in the microarray analysis of ‘Dusa’ avocado rootstocks subjected to two different levels of water stress: mild-WS (50% of Fc) and severe-WS (25% of Fc). Enrichment GO terms were obtained by Blast2GO (*p* < 0.05). BP, biological process; MF, molecular function; CC, cellular component. Black bars represent test set (mild and severe-WS) while gray bars represent the reference set.

To better understand the transcriptional responses under different levels of drought stresses, all DEGs were functionally enriched and categorized on the basis of blast sequence homologies and Gene Ontology (GO) annotations using Blast2GO software (*p* < 0.05) (Figure 5). 

DEGs were significantly grouped into the regulation of eight biological processes (BPs), eight molecular functions (MFs), and one cellular component (CC) for mild-WS treatment, and nine BPs, seven MFs, and one CC for severe-WS.

Six subcategories, belonging to the BP category, were shared by mild-WS and severe-WS, in which “cellular oxidant detoxification” (GO:0098869), followed by “cell-wall organization” (GO:0071555), “methylation” (GO:0032259), and “response to water deprivation” (GO:0009414) were among the most represented ones. These subcategories are widely associated with plant response to drought stress and subsequent cellular modification [45,46,47]. Interestingly, the “response to water deprivation” (GO:0009414) subcategory included DEGs involved in responses to the two types of stress, biotic and abiotic, i.e., calmodulin binding protein (Pa_Contig02689) [48], proteinase inhibitor (Pa_Contig00984) [49], methionine gamma-lyase (Pa_Contig00456) [50], and mitogen-activated protein kinase (Pa_Contig00290) [51]. The GO term “response to salt stress” (GO:0009651) included DEGs encoding heat-shock-like proteins (HSPs), (i.e., Pa_Contig01858, Pa_Contig02550, Pa_Contig06453, Pa_Sin_FZ03KKT01BNH1K, Pa_Sin_HA66E9C01AHKXT, Pa_Sin_HA66E9C01ALALK, Pa_Sin_HA66E9C01ARY1I, and Pa_Sin_HA66E9C01BA3Q), known to perform an essential role in plant protection against abiotic stress [52] by preventing undesired protein–protein interactions and assisting refolding of denatured proteins [53]. Among them, the I HSP class (Pa_Sin_FZ03KKT01BNH1K) was the most overexpressed gene in both mild-WS and severe-WS (Table 3).

Regarding the molecular function (MF) subcategories, “heme binding” (GO:0020037) and “peroxidase activity” (GO:0004601) were the most represented in both mild-WS and severe-WS. The ‘Dusa’ rootstock response to mild-WS involved DEGs (Pa_Contig01285, Pa_Contig00894, Pa_Contig02722, Pa_Contig00544) related to deregulation of phytohormones such as abscisic acid (ABA) (Figure 5), which plays a pivotal role in drought tolerance by inducing genes involved in dehydration resistance [54,55,56]. Other GO terms such as “endopeptidase activity” (GO:0004252; GO:0070001) “calmodulin binding” (GO:0005516), and “water transport” (GO:0006833) were associated with severe-WS. Regarding water transport, DEGs included in this subcategory were mostly repressed aquaporins (Pa_Contig00700, Pa_Contig00807, Pa_Contig00987, Pa_Contig01574, Pa_Contig02923, Pa_Contig06862), with one of them (Pa_Contig01574) included within the top 20 repressed DEGs in severe-WS (Table 3). In this regard, the differences in K_h_ and plant transpiration rate between mild-WS and severe-WS could be attributable to the downregulation of aquaporins in the latter since root aquaporins can contribute to >70% of root hydraulic conductivity [57,58].

**Figure 6:**
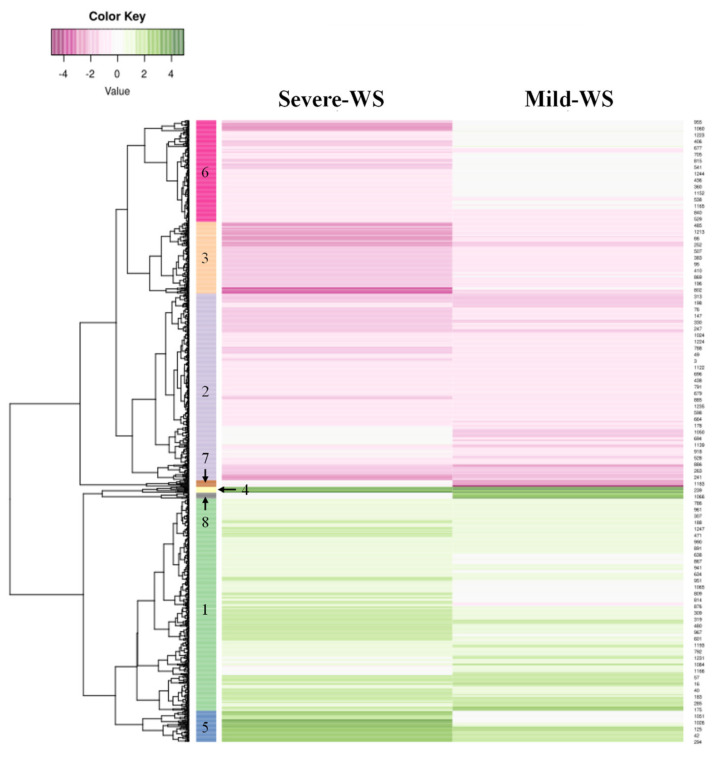
Hierarchical clustering (HCL) of differentially expressed genes (DEGs) in the microarray analysis of ‘Dusa’ avocado rootstock subjected to two different levels of water stress: mild-WS (50% of Fc) and severe-WS (25% of Fc).

Hierarchical clustering (HCL) of DEGs was performed according to the expression profiles obtained from the microarray (Figure 6). Genes were clustered into eight groups in accordance with the expression patterns observed during mild-WS and severe-WS treatments, clusters 1, 4, 5, 8 and 2, 3, 6, 7 represented contigs upregulated and downregulated, respectively. Cluster 1 was the largest group with 427 DEGs induced under both water shortage treatments. The most representative biological processes identified with GO term enriched analysis (*p* < 0.05) in this cluster comprised, among others, “response to water deprivation” (GO0009414), “negative regulation of endopeptidase activity” (GO0010951), “glutathione metabolic process” (GO0006749), “response to wounding” (GO0009611), “sodium ion transport” (GO0006814), and “serine-type endopeptidase inhibitor activity” (GO0004867), which have been extensively associated with both biotic and abiotic stress [59,60]. In particular, serine protease inhibitors play an important role in cell survival, development, and host defense, i.e., transgenic *Arabidopsis thaliana* plants overexpressing serine protease inhibitors showed higher RWC, reduced lipid peroxidation, and enhanced activity of antioxidant glutathione-*S*-transferase [61].

This cluster also grouped four genes encoding the 9-*cis*-epoxycarotenoid dioxygenase (NCED), involved in ABA biosynthesis and related to dehydration stress tolerance in *Arabidopsis* [43,62,63,64]. Interestingly, NCED genes showed higher FC values under mild-WS than in severe-WS, indicating that they are involved in the early response to water deficit. A similar expression pattern was observed for contig Pa_Contig04387 showing homology to myo-inositol-1-phoshate synthase (MIPS), which encodes a key rate-limiting enzyme involved in myo-inositol biosynthesis that plays a role in several physiological and biochemical processes such as plant immunity and hormonal regulation. Transgenic sweet potato plants overexpressing MIPS1 showed salt, drought tolerance, and stem nematode resistance, suggesting a potential use of this gene to improve resistance to biotic and abiotic stresses in plants [65].

Cluster 4 grouped only 12 DEGs that were strongly upregulated under both treatments. This group included key genes in the ‘Dusa’ response to water stress, i.e., NAC domain-containing protein 72 (Pa_Contig00313), a transcription factor linked with drought response and known to be involved in tolerance to white root rot disease in avocado [21], genes encoding a galactinol synthase (Pa_Contig02363) associated with drought tolerance [16], and several genes encoding heat-shock proteins, reported to be involved in response to both biotic and abiotic stress [52].

Cluster 5 included 63 DEGs strongly induced in severe-WS and grouped three contigs showing homology to serine carboxipeptidases-like proteins (SCPLs) (Pa_Contig06344, Pa_Contig02982, Pa_Contig01409), which are involved in regulation of defense responses against pathogen infection and oxidative stress [66]. Moreover, their overexpression improved tolerance to drought in *A. thaliana* [67]. In contrast, cluster 8 contained 13 genes showing higher induction values in mild-WS, such as ABC transporter C family member (Pa_Sin_GI32N0T02JKR74), DEAD-box ATP-dependent RNA helicase 56 isoform X2 (Pa_Sin_GI32N0T02H4DYV), pentatricopeptide repeat-containing protein At5g66520 (Pa_Sin_GI32N0T02HYARG), and tetratricopeptide TPR-1 (Pa_Sin_GI32N0T02FQL49). All of them, except for Pa_Sin_GI32N0T02FQL49, were included in the top 20 genes showing higher expression in mild-WS. Both ABC transporters and tetratricopeptide-repeat proteins are involved in ABA signaling pathways and, therefore, in the activation of genes that improve drought stress tolerance [68,69,70].

Among clusters grouping repressed contigs, Cluster 2 and 3 collected contigs that were downregulated under both water treatments; cluster 3 was the largest one with 376 DEGs of which 13 of them were represented in the top 20 list of downregulated contigs (Table 3). Cluster 2 included four pathogenesis-related protein PR-4 (Pa_Contig06278, Pa_Contig07140, Pa_Contig05982, Pa_Contig07403) and four endochitinases (Pa_Contig01261, Pa_Contig01395, Pa_Contig07157, Pa_Contig06246), previously reported to be induced by the jasmonic acid (JA) pathway known to be inhibited in response to water deprivation [71]. 

The remaining downregulated contigs were grouped into clusters 6 and 7; cluster 6 grouped those repressed in severe-WS and not affected in mild-WS, while cluster 7 brought together those repressed under mild-WS and not affected under severe-WS.

### 2.5. Modeling ‘Dusa´ Response to Different Levels of Water Stress: Linking Plant Physiology with the Root-Induced Drought-Tolerant Genes

**Figure 7:**
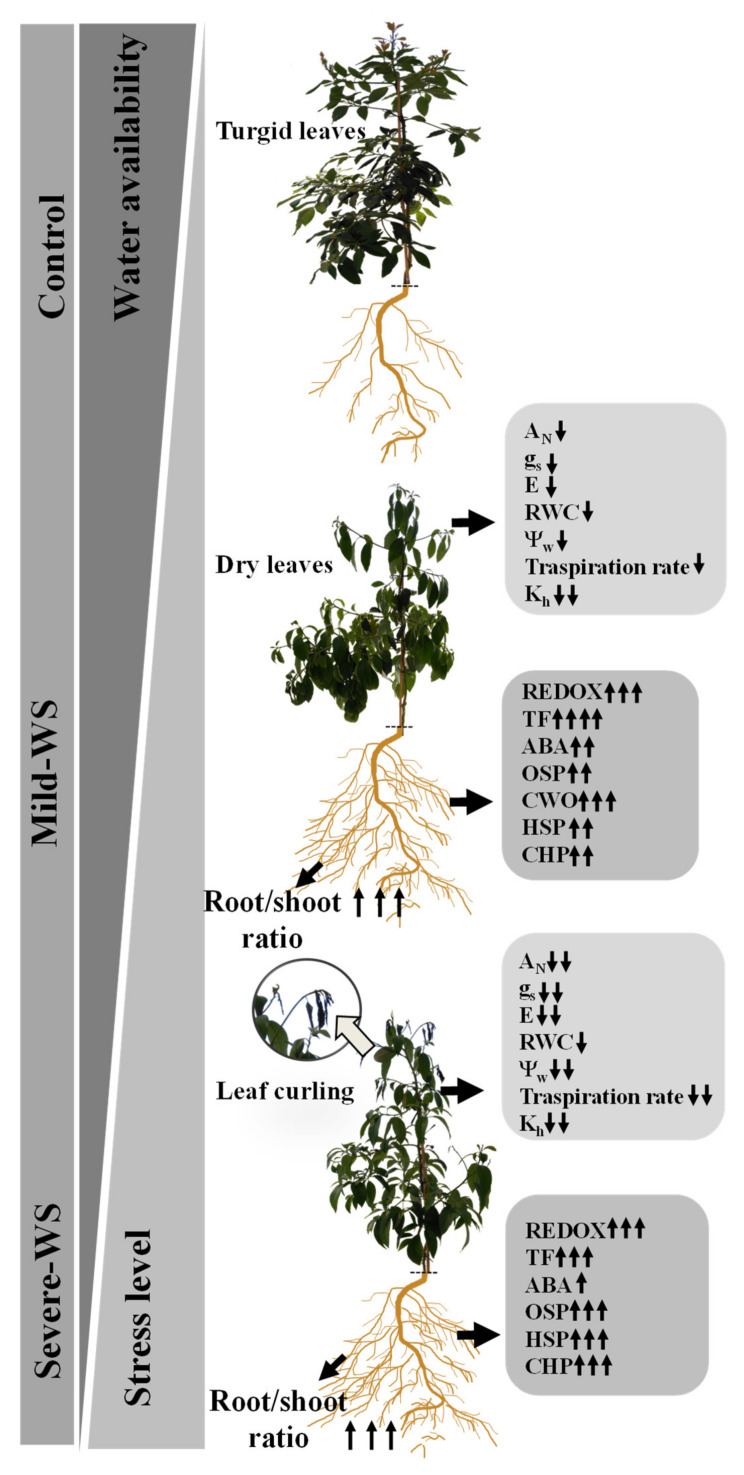
Comparative response of ‘Dusa’ avocado rootstock subjected to two different levels of water stress: mild-WS (50% of Fc) and severe-WS (25% of Fc). Physiological response to the different levels of water stress are represented with changes in net CO_2_ assimilation rate (*A*_N_), stomatal conductance (*g*_s_), transpiration rate (E), relative water content (RWC), leaf water potential (Ψ_w_), plant transpiration rate, and plant hydraulic conductance (K_h_). At a molecular level, mild and severe-WS treatment trigger the overexpression of genes encoding detoxification enzymes (REDOX), transcription factors (TFs), abscisic acid pathway (ABA), osmoprotectants (OSP), cell-wall organization (CWO), heat-shock proteins (HSPs), and chaperones (CHPs).

Plants have evolved different adaptive mechanisms to cope with water scarcity at multiple stages ranging from the molecular to whole-plant physiological level. Taking together the physiological and molecular results presented in this study, a schematic model for ‘Dusa´ rootstock in response to soil water depletion is proposed (Figure 7). At the leaf level, drought stress in ‘Dusa’ rootstock triggered water losses, lowering water potential and reducing photosynthesis, probably linked to the tight modulation of stomatal closure by ABA, which is consistent with the overexpression of genes involved in ABA biosynthetic and signaling pathways observed under both treatments in the roots.

In addition to phytohormones, numerous families of transcription factors (TF), such as NAC, MYB, and WRKY are known to be involved in signaling events associated with water stress in plants [72], playing a significant role in drought tolerance [73]. Our analysis identified 15 TF related to drought tolerance that were induced under both mild-WS and severe-WS treatments. Among them, there were five NAC domain-containing proteins, as well as four MYB and three WRKY TFs (Table 4). MYB TFs constitute one of the largest families that coordinate plant defense responses to various stresses, phytohormone signaling, and various metabolic processes [74]. Some MYB and NAC TFs have been related to lateral root growth under drought [17,19,75,76,77], which is consistent with the early increment in root biomass observed in mild-WS and severe-WS plants.

Despite the stomatal limitations of photosynthesis, the severity of the imposed stress in this study was counteracted by the induction of genes related to redox homeostasis such as those encoding peroxidases, cytochrome P450, glutathione transferases, and alterative oxidase enzymes (AOX), among others (Table 4), helping to prevent damage development and cell death as reported in previous studies [105,106,126,127,128,129,130,131,132]. In addition, a total of 18 chaperones and heat-shock proteins (HSPs) with chaperone function, probably preventing protein aggregation and denaturation during oxidative stress, were induced under both treatments, reaching higher values in severe-WS. HSPs with chaperone function included different classes such as HSP70 and HSPs below 30 kDa, all of them known to be induced in drought-tolerant plants under drought stress [139]. 

Increased drought stress intensity was not reflected in further decreases in RWC, which showed similar values under both water treatments; this could be related to the overexpression of genes involved in osmotic adjustment at the root level such as those encoding the galactinol synthase 1, galactinol–sucrose galactosyltransferase, and proline transporter (Table 4) [152,153]. This would enable water uptake and maintaining cell turgor under low water availability. In this sense, the upregulation of genes encoding xyloglucan endo-β-transglucosylases/hydrolases and expansins observed in ‘Dusa’ rootstocks after water stress (Table 4) could be linked to maintenance of cell-wall plasticity and prevention of cell turgor loss [154]. The implication of xyloglycan biosynthesis-related enzymes and expansins with drought tolerance is well documented [147,148,149,150,151].

The involvement of proteinases and proteinase inhibitor enzymes in the ‘Dusa’ response to soil water deprivation was reflected by the induction of genes encoding four serine proteases, a desumoylating isopeptidase 1 (Table 4), and four protease inhibitors. Their expression was linked to the stress severity, reaching the gene encoding a serine carboxypeptidase-like 42 with a fold-change value of 8.79 under severe-WS. Serine peptidases have been recently implicated in orchestrating the stomatal response to abiotic and biotic factors leading to enhanced water use efficiency and, therefore, drought tolerance [155]. In relation to the protease inhibitors, they have been reported to play an important role in several biological processes such as mobilization of storage proteins, regulation of endogenous enzymatic activities, modulation of apoptosis, and programmed cell death [60]. In transgenic plants, the overexpression of protease inhibitors has been associated with enhanced abiotic stress tolerance, such as water stress [49]. In addition, avocado tolerance to *Rosellinia necatrix* has been previously linked to the upregulation of protease inhibitors [21], suggesting a possible role of these proteins in the response to both biotic and abiotic stresses.

## 3. Conclusions

This study contributes to understanding the molecular mechanisms associated with drought stress response in ‘Dusa’ avocado rootstock. The microarray analysis revealed the overexpression of genes related to traits that could contribute to drought tolerance, including those involved in ABA biosynthesis, synthesis of osmoprotectants, activation of antioxidant defense, and systems repair, among others. Some of these genes have been linked to tolerance to certain biotic factors, such as fungal invasion, supporting the fact that plant responses against biotic and abiotic stress are based on common mechanisms. The molecular response, together with the ability of stressed plants to restore their physiological performance immediately after water replenishment, highlights that ‘Dusa’ avocado rootstock shows a certain degree of tolerance to water stress. Although future field trials need to be carried out using grafted plants in commercial orchards, results presented here open the possibility of using deficit irrigation as a strategy for water saving in cropping areas with limited water resources, such as the Andalusia Coast of Spain; moreover, taking into account the important role of water availability in growth of soil-borne pathogens, this water shortage could be a useful tool in soil-disease management.

## 4. Materials and Methods

### 4.1. Plant Material and Experimental Design

The study was carried out at the Institute of Agricultural Research and Training (IFAPA) (Málaga, southeastern Spain, 36°40′25″ N, 04°30′11″ W, 32 m below sea level) with 35 2 year old clonal ‘Dusa’ plants (Westfalia Estate, South Africa). Avocado plants were propagated by the Brokaw nursery (Brokaw España S.L.) using a modified Frohlich method [156] and grown in 28 L pots containing a sterilized mixture of organic substrate and sand supplemented with a slow-release fertilizer (Basacote Plus 6M, Compo Expert, Castellón, Spain). Prior to the experiment, plants were irrigated according to their needs to ensure soil wetness, and pots were covered with a black plastic to avoid soil evaporation. Once per week, plants were fertilized with an NPK solution (Kristalon Blue 17–6–18, Yara, Oslo, Norway) supplemented with iron chelate (Sequestrene^®^, Syngenta, Madrid, Spain).

The experimental design is depicted in Figure 1. Nine plants were randomly assigned to the control, in which soil moisture was maintained at field capacity (Fc) throughout the experimentation, and 26 plants were subjected to controlled substrate drying-up until they reached 50% of Fc (i.e., mild water stress, mild-WS). At this point, irrigation was restored in nine avocado plants for assessing their drought recovery response, whereas 13 plants were further desiccated until soil moisture reached 25% of Fc (i.e., severe water stress, severe-WS), which were subsequently rewatered until their initial water status was recovered (Figure 2A). 

Soil moisture was measured daily in all plants using a wet sensor (HH2 Moisture meter, Delta-T Devices. Cambridge, England). The sensor was previously calibrated for the substrate, allowing the adjustment of volumetric soil moisture (*v*/*v*) on each water treatment (mild-WS and severe-WS) in relation to the soil water holding at field capacity (Fc ~0.4 *v*/*v*). The experiment was conducted in a greenhouse under daylight illumination and semi-controlled conditions of air temperature (T) and relative humidity (RH). Photosynthetic photon flux density (PPFD), T, and RH conditions inside the greenhouse were continuously registered by a quantum sensor (Apogee SQ-110, UT, USA) and by a T/RH U23–001 HOBO^®^ Pro v2 logger (Onset Computer Corporation, MA, USA). Maximal midday values of PPFD varied between 701 and 1051 μmol·m^−2^·s^−1^, and daily T fluctuated according to external weather conditions, but its variation range inside the greenhouse was maintained between 20 ± 5 °C by an automatic cooling system and heating when necessary. The RH values inside the greenhouse were always over 33%.

Throughout the experiment, physiological measurements and root samplings were carried out at t_1_, t_2_, and t_3_. Biomass partitioning was measured in four plants from each water treatment. 

### 4.2. Physiological Measurements

Predawn (05:00–06:00 a.m.) and midday (12:00–1:00 p.m.) leaf water potential was measured using a Schölander pressure chamber (model 3005; Soil Moisture Equipment Corporation, Santa Barbara, CA, USA). On each plant, one mature fully developed leaf per plant close to the main stem was measured following the recommendations made by Hsiao [157]. Maximal photochemical efficiency of PSII (*F*v/*F*m) was also measured at predawn using a Pulse Amplitude Modulation (PAM-2000) fluorometer (Heinz Walz GmbH, Effeltrich, Germany). Relative leaf water content (RWC), the specific leaf mass area (LMA), and relative chlorophyll content (SPAD index) were measured only at t_1_ in the same plants as for leaf water potential. For RWC determinations, leaf discs (2 cm^2^) were taken at midday and weighed to obtain fresh weight (F_W_), before being immediately imbibed on distilled water for 24 h at 5 °C in darkness for obtaining turgid weight (T_W_). Afterward, samples were oven-dried at 80 °C for 48 h to get dry weight (D_W_). RWC was calculated as follows:

RWC (%) = ((F_W_ − D_W_)/(T_W_ − D_W_)) × 100.

The specific LMA was calculated as the ratio between disc Dw and disc area (g·cm^−2^). LMA values were used to translate leaf dry biomass into plant foliar area. Leaf gas exchange was measured in one mature exposed leaf per plant at midday (11:00 a.m.–2:00 p.m.) at t_1_ and t_2_. Measurements were done with an open portable photosynthesis system (model LI-6400, LI-COR, NE, USA) equipped with an LED light source (6400–02B) coupled to a sensor head/IRGA, and with a CO_2_ mixer (6400–01). Settings for measurements were as follows: flow rate, 500 mL·min^−1^; CO_2_ partial pressure, 400 ppm; photosynthetic photon flux density, 1000 µmol·m^−2^·s^−1^; leaf temperature, ~20 °C; relative humidity, ~50% (vapor pressure deficit ~1.4 kPa). Net CO_2_ assimilation rates (*A*_N_), stomatal conductance (*g*_s_), and transpiration rates (*E*) were estimated using the equations of Von Caemmerer and Farquhar [158]. The SPAD index was nondestructively measured at midday on one leaf per plant using a handheld SPAD 502 meter (Minolta, Osaka, Japan). This index provides an estimation of leaf chlorophyll content [159]. On each plant, averaged SPAD values were calculated from three readings along the leaf. Plant hydraulic conductance (K_h_, mmol H_2_O·m^−2^·s^−1^·MPa^−1^) was calculated using Poiseuille’s law analogy for the soil–plant–atmosphere continuum [160].

E_plant_ = (Ψ_MD_ − Ψ_PD_) × K_h_,

where Ψ_MD_ and Ψ_PD_ are the midday and predawn water potential (MPa), and E_plant_ is the transpiration rate expressed as mmol H_2_O·m^−2^·s^−1^.

Plant transpiration was calculated as the weight decrease between predawn and midday (pots were covered with plastic to avoid evaporation). Plant foliar area was calculated to convert water uptake into transpiration rate.

After completing the set of physiological measurements, four plants from each water treatment were removed from pots and divided into leaves, stems, and roots. Obtained samples were put in paper envelopes and placed in an oven at 80 °C until reaching a constant weight that was assumed to be dry weight.

### 4.3. RNA Extraction

Avocado roots from control, mild-WS, and severe-WS plants were harvested at t_1_ and t_2_. Three biological replicates per timepoint, in which each biological replicate consisted of three plants (*n* = 9), were used for RNA extraction in each experimental group. RNA from ground root tissue was extracted using the CTAB extraction method [161] with modification described by Zumaquero et al. [21]. RNA parameters and integrity were checked using a NanoDrop® ND-1000 (Nanodrop Technologies Inc., Wilmington, DE, USA) spectrophotometer based on the A260/280 and A260/230 wavelength ratios and running samples on a 2% agarose gel. RNA samples were treated with a DNase treatment with 1 U of RNase-free DNase (Thermo Scientific, Life Technologies Inc., Carlsbad, CA, USA), 1 μL of 10× reaction buffer with MgCl_2_, 1 μg of RNA, 0.5 μL of RiboLock RNase Inhibitor (Thermo Scientific Inc., CA, USA), and diethylpyrocarbonate-treated water to a final volume of 10 μL in all RNA samples. The mixture was incubated according to the manufacturer’s instructions at 37 °C for 45 min followed by the addition of 1 μL of 50 mM EDTA and incubation at 65 °C for 10 min according to the manufacturer’s instructions.

### 4.4. Microarray Analysis

Microarray hybridizations were carried out using a custom microarray (GEO accession GPL21856) as previously described by Reeksting et al. [20]. For mild-WS plants, three biological replicate hybridizations of water treatment vs. control samples were performed, while two biological replicate hybridizations of water treatment vs. control samples were performed for severe-WS. Microarray data were statistically analyzed using the LIMMA (linear models for microarray data) package in the R version 3.1.0 environment (R Foundation for Statistical Computing) as described in Reeksting et al. [20]. The *p*-values were corrected for multiple testing by the false discovery rate (FDR) method. To determine concordance between biological replicates, a standard pairwise Pearson correlation (*r*) was performed using normalized M-values. In this study, targets were defined as differentially expressed genes (DEG) if the adjusted *p*-value was less than or equal to 0.05 (*p* ≤ 0.05) and the log_2_ ratio ≥1, or the log_2_ ratio ≤1. The data from this experiment are available from the NCBI Gene Expression Omnibus under accession number GSE151051.

### 4.5. Functional Annotation and Clustering

The software suite Blast2GI (B2G: http://www.blast2go.com. accessed on 1 September 2020) was used to assign Gene Ontology (GO) terms describing biological processes, molecular functions, and cellular components, as well as to perform functional annotation and functional enrichment. A reduction to the most specific terms was applied using the software default parameters and a *p*-value cutoff of 0.05. Genes with similar expression profiles across all three biological samples were identified by a hierarchical clustering analysis of their expression values. The results were then processed with the function hclust, from the stats package (R Core Team. 2017), to calculate Person correlation and to perform a linkage analysis. The dendrograms were plotted as a heatmap with the heatmap.2 function from gplots [162], and an R color Brewer [163] schema was applied in order to facilitate visualization. A unique expression profile was used to define each distinctive cluster of contigs (represented by the dendrogram rows) using cutree, from the stats package (R Core Team. 2017).

### 4.6. Quantitative Real-Time PCR

Quantitative real-time PCR (qRT-PCR) was used to validate microarray results. Single-stranded cDNA was synthesized using an iScript Reverse Transcription Supermix kit (Bio-Rad Laboratories Inc., California, USA). DNA contamination was checked by PCR using gene specific primers F3H-F (5′–TCTGATTTCGGAGATGACTCGC–3′) and F3H-R (5′–TGTAGACTTGGGCCACCTCTTT–3′), which flank an intron of the flavanone 3-hydroxylase (F3H) gene. PCR amplifications were carried out using first-strand cDNA as the template as previously described by Engelbrecht and van den Berg [164]. 

Primer sequences for amplification of the endogenous control gene (actin gene) and 13 avocado genes were designed using Primer 3 software (http://bioinfo.ut.ee/primer3–0.4.0/. accessed on 1 September 2020) (Table S1). qRT-PCR was performed following the methodology detailed in Zumaquero et al. [21]. All reactions were carried out in triplicate, and relative quantification of the expression levels was analyzed using the comparative Ct method [165]. 

### 4.7. Statistical Analysis

Statistical analyses were performed using the analytical software STATISTICA 7 (StatSoft Inc., OK, USA). Analysis of variance (ANOVA) was used for assessing significant differences (*p* < 0.05) among treatments in physiological variables. Normality and homogeneity assumptions for ANOVA were tested using the Kolmogorov–Smirnov and the Cochran’s C test, respectively. Pairwise comparisons of the means were done using Fisher’s least significant difference (LSD) test. Statistics for qRT-PCR data were tested using Student’s *t*-test.

## Figures and Tables

**Table 1:** Plant dry biomass parameters and leaf mass area (LMA) from ‘Dusa’ avocado rootstock under two different levels of water stress: mild-WS (50% of Fc) and severe-WS (25% of Fc). The table shows mean values (±SE; *n* = 4) under stress and after the corresponding recovery. Different letters indicate significant differences among treatments (*p* < 0.05, ns: no statistical differences).

	Control		Mild-WS		Severe-WS		Mild-WSRecovery		Severe-WSRecovery	
*Plant dry biomass* (g)	567.94 ± 63.76	ns	504.08 ± 64.02	ns	554.43 ± 38.84	ns	572.93 ± 77.63	ns	571.70 ± 30.88	ns
*Leaf dry biomass* (%)	29.09 ± 2.74	ns	21.26 ± 2.18	ns	23.30 ± 2.57	ns	29.87 ± 1.96	ns	23.09 ± 2.57	ns
*Stem dry biomass* (%)	43.69 ± 1.45	ns	41.46 ± 2.07	ns	39.00 ± 2.27	ns	38.91 ± 0.82	ns	39.30 ± 0.37	ns
*Root dry biomass* (%)	27.22 ± 2.60	^b^	37.28 ± 0.95	^a^	37.70 ± 2.45	^a^	31.22 ± 2.42	^ab^	37.61 ± 2.60	^a^
*Root /shoot ratio*	0.38 ± 0.05	^b^	0.60 ± 0.02	^a^	0.61 ± 0.06	^a^	0.46 ± 0.05	^ab^	0.61 ± 0.06	^a^
*Leaf mass area* (g·m^−2^)	98.76 ± 3.09	^ab^	90.05 ± 1.52	^b^	74.17 ± 5.50	^c^	104.17 ± 6.78	^a^	86.90 ± 6.92	^bc^

**Table 2:** qRT-PCR and microarray expression data of selected contigs from ‘Dusa’ avocado rootstock under two different levels of water stress: mild-WS (50% of Fc) and severe-WS (25% of Fc). Numbers in bold indicate statistically significant results (*p* < 0.05).

		Mild-WS		Severe-WS	
Annotation	Contig	Microarray FC	q-RT FC	Microarray FC	q-RT FC
PR5	Pa_Contig01462	2.80	4.06	6.10	7.24
Profilin 1 isoform 1	Pa_Contig02273	1.07	1.39	−1.10	−1.22
Protease inhibitor II	Pa_Contig03907	−1.20	−1.68	2.12	4.31
Alcohol dehydrogenase b	Pa_Sin_GI32N0T02IUGTU	1.04	1.61	−1.05	−2.00
LRR resistance PLP	Pa_Contig01244	1.06	1.39	−1.12	−1.22
Trypsin inhibitor	Pa_Contig04097	−2.39	−1.18	1.16	1.51
Sucrose synthase	Pa_Contig00004	−1.83	−2.22	−1.54	−1.44
Phenylalanine ammonia-lyase (PAL)	Pa_Contig00410	−1.00	−1.59	−1.41	−1.51
Chalcone synthase	Pa_Contig00619	−1.50	−3.54	−3.02	−6.10
Xyloglucan endotransglucosylase hydrolase	Pa_Contig00751	−2.04	−4.63	−2.71	−7.94
Defensin j1-2-like	Pa_Contig04185	−1.99	−2.32	−2.04	−1.33
Lipoxygenase (LOX)	Pa_Contig04337	−1.74	−2.29	−1.85	−4.15
PR4	Pa_Contig06278	−3.28	−2.67	−3.78	−5.60

**Table 3:** Top 20 induced and top 20 repressed differentially expressed genes (DEGs) from ‘Dusa’ avocado rootstock subjected to two different levels of water stress: mild-WS (50% of Fc) and severe-WS (25% of Fc) (NA, nonannotated; FC, fold change).

Mild-WS			Severe-WS		
Name	Description	FC	Name	Description	FC
Pa_Sin_FZ03KKT01BNH1K	18.1 kDa class I heat-shock protein-like	28.94	Pa_Sin_FZ03KKT01BNH1K	18.1 kDa class I heat-shock protein-like	23.07
Pa_Sin_HA66E9C01BSEGX	Splicing factor SF3a60 homolog	22.31	Pa_Contig05542	NA	21.08
Pa_Contig03188	Transmembrane protein TauE-like	19.52	Pa_Sin_GI32N0T02G3V3U	DExH-box ATP-dependent RNA helicase DExH3	14.81
Pa_Contig03520	Transmembrane protein TauE-like	18.46	Pa_Contig03188	Transmembrane protein TauE-like	13.97
Pa_Sin_GI32N0T02J3CHK	PREDICTED: uncharacterized protein LOC103961965	14.94	Pa_Sin_HA66E9C01AIWJ3	25.3 kDa heat-shock protein	12.05
Pa_Contig02835	Probable nucleoredoxin 2	14.42	Pa_Contig03520	Transmembrane protein TauE-like	11.97
Pa_Contig05542	NA	13.94	Pa_Sin_GI32N0T02IBYBH	Phospholipase D beta 1	11.80
Pa_Contig00313	NAC domain-containing protein 72	12.71	Pa_Contig04544	Hypothetical protein CKAN_02127100	11.75
Pa_Contig00357	CTP synthase-like isoform X1	10.36	Pa_Contig02363	galactinol synthase 1	11.10
Pa_Contig02363	Galactinol synthase 1	9.48	Pa_Sin_HA66E9C01ARY1I	17.9 kDa class II heat-shock protein-like	10.71
Pa_Sin_GI32N0T02JKR74	ABC transporter C family member 3	9.04	Pa_Sin_GI32N0T02IZT2Y	Zinc finger MYM-type protein 1-like protein	10.24
Pa_Sin_GI32N0T02H4DYV	DEAD-box ATP-dependent RNA helicase 56 isoform X2	8.56	Pa_Contig00313	NAC domain-containing protein 72	10.18
Pa_Sin_HA66E9C01B0D6Q	Redoxin	7.93	Pa_Sin_HA66E9C01AOOV3	ATP synthase subunit G mitochondrial-like	10.00
Pa_Sin_GI32N0T02HYARG	Pentatricopeptide repeat-containing protein At5g66520	7.75	Pa_Contig00357	CTP synthase-like isoform X1	9.80
Pa_Contig03826	Transcription factor SPATULA-like	7.67	Pa_Contig05359	Wound-responsive family protein	9.53
Pa_Contig06401	CTP synthase-like	7.56	Pa_Contig04498	NA	9.48
Pa_Contig04336	Trinucleotide repeat-containing gene 18 protein	7.15	Pa_Sin_GI32N0T02JFLZB	Pentatricopeptide repeat-containing protein At1g08070	8.80
Pa_Contig03089	Snakin-2	7.07	Pa_Contig06344	Serine carboxypeptidase-like 42	8.79
Pa_Sin_HA66E9C01ARY1I	17.9 kDa class II heat-shock protein-like	6.92	Pa_Contig02835	Probable nucleoredoxin 2	8.38
Pa_Sin_GI32N0T02JL4B4	9-*cis*-epoxycarotenoid dioxygenase	6.75	Pa_Sin_HA66E9C01AKONC	Pyrophosphatase domain-containing protein	8.17
Pa_NA_RC_Contig06917	NA	−30.10	Pa_Sin_HA66E9C01AZE26	Major pollen allergen Bet v 1-F/I	−15.16
Pa_Sin_GI32N0T02JH50H	Photosystem I P700 apoprotein A1	−19.40	Pa_Contig07385	Cysteine peptidase, asparagine active site-containing protein	−14.04
Pa_Sin_GI32N0T02I198R	DExH-box ATP-dependent RNA helicase DExH3	−18.78	Pa_Contig07552	Peptidase_C1 domain-containing protein/Inhibitor_I29	−11.27
Pa_Sin_HA66E9C01A762Y	Superoxide dismutase [Mn], mitochondrial	−9.61	Pa_Sin_GI32N0T02GHCO6	Plasma membrane ATPase 1	−11.17
Pa_Sin_HA66E9C01ABCHY	Protein unc-13 homolog	−8.37	Pa_Sin_GI32N0T02I0NSB	Pyruvate decarboxylase 2	−10.98
Pa_Sin_GI32N0T02IB55S	Hypothetical protein	−7.29	Pa_Contig03628	Peptidoglycan-binding Lysin subgroup	−10.89
Pa_NA_RC_Contig06574	NA	−7.22	Pa_Contig01574	TIP protein	−9.40
Pa_Sin_HA66E9C01BUD7H	Aspartate/other aminotransferase	−6.45	Pa_Contig02013	Stellacyanin-like protein	−9.09
Pa_Sin_GI32N0T02F883Q	Acyl-protein thioesterase, putative	−6.26	Pa_Contig00293	Putative laccase 9	−9.03
Pa_Sin_GI32N0T02J1G6J	SusD/RagB family nutrient-binding lipoprotein	−5.90	Pa_Sin_HA66E9C01AN9EE	Peroxidase 3-like	−8.82
Pa_NA_F_contig07053	NA	−5.84	Pa_Contig06873	Hypothetical protein CKAN_01558700	−8.71
Pa_Contig03714	l-Idonate 5-dehydrogenase	−5.46	Pa_Sin_GI32N0T02GK4GX	Putative senescence-associated protein	−8.64
Pa_NA_RC_Contig07246	Hypothetical protein PHALS_14482	−5.36	Pa_Contig05711	Senescence-specific cysteine protease SAG39-like	−8.56
Pa_Contig01285	Pathogenesis-related protein 1-like protein	−5.19	Pa_Sin_HA66E9C01BC645	HSP20-like chaperone	−8.47
Pa_Contig00582	BTB/POZ and TAZ domain-containing protein 1-like	−5.10	Pa_Sin_GI32N0T02IPVS0	HSP70-like protein	−8.26
Pa_Sin_GI32N0T02FFZXN	Patatin/phospholipase A2-related	−5.02	Pa_Contig03714	l-Idonate 5-dehydrogenase	−8.17
Pa_Sin_GI32N0T02GE7BG	Pyruvate decarboxylase 1	−4.84	Pa_Sin_GI32N0T02JFZ2K	LURP1-like domain-containing protein	−8.11
Pa_Contig06521	Hypothetical protein VOLCADRAFT_107374	−4.69	Pa_Contig05100	14 kDa proline-rich protein DC2.15-like	−7.89
Pa_Contig01605	36.4 kDa proline-rich protein	−4.69	Pa_Sin_GI32N0T02J1LXF	Pentatricopeptide repeat-containing protein At5g18475	−7.43
Pa_Contig01261	Basic endochitinase-like protein	−4.64	Pa_Contig05579	14 kDa proline-rich protein DC2.15-like	−7.36

**Table 4:** Water stress-related differentially expressed genes (DEGs) from ‘Dusa’ avocado rootstocks subjected to two different levels of water stress: mild-WS (50% of Fc) and severe-WS (25% of Fc) (FC, fold change).

Sequence name	Description	Function	Mild-WS FC	Severe-WS FC	References
*Transcription factor*					
Pa_Contig00978	B-box zinc finger protein 32	Transcription factor	2.50	2.73	[78,79,80]
Pa_Contig00204	Zinc finger CCCH domain-containing protein 20	Transcription factor	2.54	2.66	[78,79,80]
Pa_Contig04595	Zinc finger protein ZAT10	Transcription factor	2.45	2.85	[78,79,80]
Pa_Sin_GI32N0T02FVARP	Heat stress transcription factor B-2a	Transcription factor	3.33		[81]
Pa_Sin_GI32N0T02J04TC	Heat stress transcription factor B-3	Transcription factor	2.44	3.13	[81]
Pa_Sin_GI32N0T02GJ71A	Heat stress transcription factor C-1	Transcription factor	4.41	5.44	[81]
Pa_Contig00660	Homeobox-leucine zipper protein HAT5	Transcription factor	4.51	2.32	[82]
Pa_Contig01191	NAC domain-containing protein 2	Transcription factor	3.88	4.15	[83,84,85,86,87]
Pa_Contig03450	NAC domain-containing protein 2	Transcription factor	3.67	5.30	[83,84,85,86,87]
Pa_Contig00313	NAC domain-containing protein 72	Transcription factor	12.71	10.18	[83,84,85,86,87]
Pa_Contig07055	NAC domain-containing protein 82-like protein	Transcription factor	1.88	2.60	[83,84,85,86,87]
Pa_Contig07561	NAC domain-containing protein 82-like protein	Transcription factor	1.94	2.57	[83,84,85,86,87]
Pa_Contig03801	Probable WRKY transcription factor 31	Transcription factor		2.05	[88]
Pa_Contig04109	Probable WRKY transcription factor 48	Transcription factor	1.33	2.48	[88]
Pa_Contig03985	Transcription factor MYB1R1	Transcription factor	2.15	2.64	[89]
Pa_Contig05714	Transcription factor MYBS3	Transcription factor, Response to hormones	2.06	1.99	[89]
Pa_Contig05191	Trihelix transcription factor ASIL2	Transcription factor	1.69	2.36	[90,91,92]
*Hormonal regulation*					
Pa_Sin_GI32N0T02JL4B4	9-*cis*-Epoxycarotenoid dioxygenase	Abscisic acid signaling pathway	6.75	2.34	[62,63,93,94,95]
Pa_Sin_GI32N0T02HK1RI	9-*cis*-Epoxycarotenoid dioxygenase NCED1	Abscisic acid signaling pathway	5.71	1.55	[62,63,93,94,95]
Pa_Sin_HA66E9C01AOCH8	Putative 9-*cis*-epoxycarotenoid dioxygenase	Abscisic acid signaling pathway	4.29	1.70	[62,63,93,94,95]
Pa_Contig04541	Abscisic acid-insensitive 5-like protein 5	Abscisic acid signaling pathway	2.30	1.70	[96,97]
Pa_Contig01488	Probable protein phosphatase 2C 24	Abscisic acid signaling pathway	4.19	2.12	[98,99]
Pa_Contig04387	Myo-inositol-1-phosphate synthase	Stress signaling	5.97	3.80	[100,101,102,103]
*Redox homeostasis*					
Pa_Contig00910	Ubiquinol oxidase 2	Alternative oxidase (AOX) activity	2.52	3.78	[104]
Pa_Contig02586	Glutathione *S*-transferase 23-like	Glutathione transferase activity	2.32	3.50	[105,106,107]
Pa_Contig05480	Probable glutathione *S*-transferase	Glutathione transferase activity	2.14	1.88	[105,106,107]
Pa_Contig01550	Probable glutathione *S*-transferase parA	Glutathione transferase activity	2.14	1.42	[105,106,107]
Pa_Contig01827	Putative glutathione *S*-transferase	Glutathione transferase activity	4.40	2.62	[105,106,107]
Pa_Contig02245	Alcohol dehydrogenase superfamily, zinc-type	Oxidoreductase activity	2.01	2.26	[108]
Pa_Sin_GI32N0T02JXR49	Amine oxidase	Oxidoreductase activity	2.13	2.12	[109,110]
Pa_Sin_HA66E9C01AHBP7	Amine oxidase	Oxidoreductase activity	2.09	2.37	[109,110]
Pa_Sin_GI32N0T02HAOKJ	Cytokinin dehydrogenase 7 isoform X2	Oxidoreductase activity	2.04		[111,112,113]
Pa_Sin_GI32N0T02H5ZVO	Lipoxygenase 6	Oxidoreductase activity		2.04	[114,115,116]
Pa_Contig01521	NADP-dependent glyceraldehyde-3-phosphate dehydrogenase	Oxidoreductase activity	2.44	2.03	[117,118]
Pa_Sin_GI32N0T02IWQT9	Probable cinnamyl alcohol dehydrogenase 6	Oxidoreductase activity, lignin biosynthesis		2.68	[119,120,121]
Pa_Contig04086	Sorbitol dehydrogenase	Oxidoreductase activity	3.03	3.47	[122,123]
Pa_Contig03274	Thioredoxin-like protein CXXS1	Oxidoreductase activity		2.17	[124,125]
Pa_Contig01894	Corytuberine synthase (Cytochrome P450)	Oxidoreductase activity	2.28	1.35	[126,127,128]
Pa_Contig01546	Cytochrome P450 CYP72A219-like protein	Oxidoreductase activity	1.71	2.07	[126,127,128]
Pa_Contig01573	Cytochrome P450 714C2-like	Oxidoreductase activity	3.88	3.22	[126,127,128]
Pa_Contig01652	Cytochrome P450 71A1	Oxidoreductase activity	2.37	3.52	[126,127,128]
Pa_Contig07139	Cytochrome P450 71A1	Oxidoreductase activity	2.34	3.11	[126,127,128]
Pa_Contig07325	Cytochrome P450 71A1	Oxidoreductase activity	2.04	3.00	[126,127,128]
Pa_Contig07667	Cytochrome P450 71A1	Oxidoreductase activity	2.36	3.16	[126,127,128]
Pa_Contig00616	Cytochrome P450 89A2	Oxidoreductase activity	3.03	2.80	[126,127,128]
Pa_Contig04644	Cytochrome P450 89A2	Oxidoreductase activity	5.79	3.53	[126,127,128]
Pa_Sin_HA66E9C01B0D6Q	1-Cys peroxiredoxin isozyme	Peroxidase activity	7.93		[129]
Pa_Sin_GI32N0T02JBZZB	Peroxiredoxin Q	Peroxidase activity		2.25	[129]
Pa_Contig05152	Cationic peroxidase 1-like	Peroxidase activity	2.01		[130,131,132]
Pa_Contig06649	Cationic peroxidase 1-like	Peroxidase activity	2.13	5.48	[130,131,132]
Pa_Contig04553	Peroxidase 12-like	Peroxidase activity		2.01	[130,131,132]
*Protease and protease inhibitor activity*					
Pa_Contig01409	Serine carboxypeptidase-like 42	Intracellular turnover of proteins	2.69	5.98	[67,133]
Pa_Contig02982	Serine carboxypeptidase-like 42	Intracellular turnover of proteins	1.87	6.32	[67,133]
Pa_Contig06344	Serine carboxypeptidase-like 42	Intracellular turnover of proteins	2.19	8.79	[67,133]
Pa_Contig03889	Desumoylating isopeptidase 1	Post-translational mechanism in respond to stress	1.71	2.02	[134]
Pa_Contig02540	Kunitz trypsin inhibitor 2	Protease inhibitor, wound and herbivores response	1.40	2.10	[59]
Pa_Contig00984	Cysteine proteinase inhibitor 12-like	Proteinase inhibitor	1.94	2.36	[49,61,135,136]
Pa_Contig05072	Proteinase inhibitor	Proteinase inhibitor	1.44	2.47	[49,61,135,136]
Pa_NA_RC_Contig07158	Proteinase inhibitor I3	Proteinase inhibitor	1.42	3.05	[49,61,135,136]
Pa_Contig03565	Subtilisin-like protease SBT3.17	Serine protease, plant defense response		2.68	[137,138]
*Chaperone and heat shock proteins*					
Pa_Contig03398	Chaperone protein ClpB1	Chaperone activity	3.33	4.30	[139]
Pa_Sin_GI32N0T02HS82J	Chaperone protein ClpB1	Chaperone activity	3.56	5.20	[139]
Pa_Sin_GI32N0T02HS9YT	Chaperone protein ClpB1	Chaperone activity	3.70	6.05	[139]
Pa_Contig03328	Chaperone protein dnaJ 11	Chaperone activity	2.47	2.68	[139]
Pa_Sin_HA66E9C01AV9KD	Chaperonin-like RbcX protein 2	Chaperone activity	2.86	3.94	[139]
Pa_Sin_GI32N0T02GU01I	Chaperonin-like RbcX protein 2	Chaperone activity		3.65	[139]
Pa_Sin_HA66E9C01BZUZG	15.7 kDa heat-shock protein, peroxisomal	Heat-shock protein activity	2.12	3.43	[139]
Pa_Sin_HA66E9C01AHKXT	17.3 kDa class II heat-shock protein	Heat-shock protein activity	5.67	5.93	[139]
Pa_Contig01858	17.8 kDa class I heat-shock protein-like	Heat-shock protein activity	3.94	5.70	[139]
Pa_Sin_HA66E9C01ARY1I	17.9 kDa class II heat-shock protein-like	Heat-shock protein activity	6.92	10.71	[139]
Pa_Sin_FZ03KKT01BNH1K	18.1 kDa class I heat-shock protein-like	Heat-shock protein activity	28.94	23.07	[139]
Pa_Sin_HA66E9C01AIWJ3	25.3 kDa heat-shock protein	Heat-shock protein activity	6.14	12.05	[139]
Pa_Contig02550	Class I heat-shock-like protein	Heat-shock protein activity	3.23	5.38	[139]
Pa_Sin_GI32N0T02I40L4	Class I heat-shock-like protein	Heat-shock protein activity	1.54	3.31	[139]
Pa_Sin_HA66E9C01AFKWO	Heat-shock 70 kDa protein	Heat-shock protein activity		3.60	[139]
Pa_Sin_GI32N0T02GPO75	Heat-shock 70 kDa protein 15-like	Heat-shock protein activity	4.71		[139]
Pa_Sin_GI32N0T02J33GV	Heat-shock 70 kDa protein 17	Heat-shock protein activity	3.30		[139]
Pa_Contig00041	Heat-shock cognate 70 kDa protein 2	Heat-shock protein activity	1.64	2.02	[139]
Pa_Sin_GI32N0T02FE65Z	Heat-shock protein	Heat-shock protein activity	3.59	5.30	[139]
Pa_Contig04262	Heat-shock protein 70	Heat-shock protein activity	2.05	2.63	[139]
Pa_Contig00058	Heat-shock protein 83	Heat-shock protein activity	3.74	4.25	[139]
Pa_Contig05589	Small heat-shock protein	Heat-shock protein activity	1.82	2.40	[139]
*Osmoprotectant*					
Pa_Contig02363	Galactinol synthase 1	Galactose metabolism	9.48	11.10	[140,141,142]
Pa_Contig04773	Galactinol synthase 1-like	Galactose metabolism	5.25	6.49	[140,141,142]
Pa_Contig00418	Probable galactinol–sucrose galactosyltransferase 2	Myo-inositol and raffinose synthesis	2.15	2.83	[100,101,102,103]
Pa_Contig02227	Probable galactinol–sucrose galactosyltransferase 2	Myo-inositol and raffinose synthesis	2.14	2.30	[100,101,102,103]
Pa_Sin_GI32N0T02GAY0V	Proline transporter 2-like	Proline metabolic process	2.65	3.14	[143,144,145]
Pa_Contig05170	Beta-fructofuranosidase, soluble isoenzyme I-like	Sucrose metabolic process	4.03	5.32	[146]
*Cell-wall organization*					
Pa_Contig05067	Expansin-like A2	Cell-wall organization	2.41	1.55	[147,148]
Pa_Contig00733	Probable xyloglucan endotransglucosylase/hydrolase protein 23	Cell-wall organization	4.64	5.16	[149,150,151]
Pa_Contig01176	Probable xyloglucan endotransglucosylase/hydrolase protein 27	Cell-wall organization	2.14	1.86	[149,150,151]

## Data Availability

All data generated or analyzed during this study are included in this published article. The data from this study are available from the NCBI Gene Expression Omnibus under accession number GSE151051.

## Supplementary Materials

The following are available online at www.mdpi.com/article/10.3390/plants10102077/s1. Table S1. qRT-PCR primer sequences used in this study.

## Authors’ Information

M.-O.G. is a graduate student at the PhD Program of Advanced Biotechnology, University of Málaga.
