# Supplementary material for: Physiological and Molecular Responses of ‘Dusa’ Avocado Rootstock to Water Stress: Insights for Drought Adaptation
Source: Plants (Basel). 2021 Sep 30;10(10):2077. doi: 10.3390/plants10102077 (PMC8537572; doi:10.3390/plants10102077)
Supplement: Supplementary file 1 [file plants-10-02077-s001.zip › plants-1384703-S1.pdf]

Supplementary Table S1. qRT-PCR primer sequences used in this study.

Description	Contig/ GenBank ID	Amplicon Tm (°C)	Amplicon size (bp)	Primer sequences (forward/reverse) [5′-3′]
Actin	Pa_Contig00256	58	104	CCAAGCAGCATGAAGATAAAGGT<br>CACATCTGTTGGAAGGTGCTC
PR5	Pa_Contig01462	60	135	GACACCAGCCTAGTCGATGG<br>TCCATCCGGTGCTGTCAATC
Profilin 1 isoform 1	Pa_Contig02273	58.5	110	TTCGGTATCTATGATGAG<br>ACGATATGACATTCAATAG
Protease inhibitor II	Pa_Contig03907	60	122	GATGGCGCTGGTCAAGAAGA<br>AATGCTTGCTGGGAGTCTCG
Alcohol dehydrogenase b	Pa_Sin_GI32N0T02IUGTU	60	103	TGGTTGCTGTGCTAGTAGGT<br>TTCCAAAGAAAGTTCCCTTGAGAG
LRR resistance PLP	Pa_Contig01244	60	99	AGCACCCGTTCTTGAAGGAAA<br>CGGAGTCTGAAACCTCAAGC
Trypsin inhibitor	Pa_Contig04097	60	101	TTCAATGTGCTTCTGGTGGCT<br>TTCGCCATCATCCCATCACC
Sucrose synthase	Pa_Contig00004	57	98	CATACATCAAACCGTGAGATCCA<br>TCAAGATGACCTGTGAAGCA
Phenylalanine ammonia-lyase (PAL)	Pa_Contig00410	60	79	TTGATGACCCATGCAGCTGT<br>CCATTGATCAGCGCGTGTTC
Chalcone synthase	Pa_Contig00619	60	103	AGGCAAGACATGGTGGTGG<br>CCAGGTGGGTGATCTTGGAC
Xyloglucan endotransglucosylase hydrolase fam. prot.	Pa_Contig00751	60	97	TCCTCAGCGCATCATCTTCTC<br>CATGGGCTGCTTGTTAGGGA
Defensin j1-2-like	Pa_Contig04185	60	145	GCTGCTCGTTCTTCTTCTCCT<br>ATGGAAGCCTTCAATCCTGCA
Lipoxygenase (LOX)	Pa_Contig04337	60	72	CGAACATGCCAGTGGAGG<br>ACATTGCAGACTCAGGCC
PR4	Pa_Contig06278	60	136	TTGACACTGACGGAAGAGG<br>TGTAAATCAGTCGCCGCAG
